# Taxonomy of the *Trichophyton mentagrophytes/T. interdigitale* Species Complex Harboring the Highly Virulent, Multiresistant Genotype *T. indotineae*

**DOI:** 10.1007/s11046-021-00544-2

**Published:** 2021-04-13

**Authors:** Chao Tang, Xue Kong, Sarah A. Ahmed, Rameshwari Thakur, Anuradha Chowdhary, Pietro Nenoff, Silke Uhrlass, Shyam B. Verma, Jacques F. Meis, Hazal Kandemir, Yingqian Kang, G. Sybren de Hoog

**Affiliations:** 1 Key Laboratory of Environmental Pollution Monitoring and Disease Control, Ministry of Education of Guizhou & Key Laboratory of Medical Microbiology and Parasitology, School of Basic Medical Sciences, Guizhou Medical University, Guiyang, China; 2 Center of Expertise in Mycology of Radboud University Medical Center, Canisius Wilhelmina Hospital, Nijmegen, The Netherlands; 3 Department of Mycology, Institute of Dermatology, Chinese Academy of Medical Science and Peking Union Medical College, Nanjing, 0210042 China; 4 Department of Dermatology and Microbiology, Muzaffarnagar Medical College and Hospital, Chaudhary Charan Singh University, Meerut, India; 5 Department of Medical Mycology, Vallabhbhai Patel Chest Institute, University of Delhi, Delhi, India; 6 Laboratory for Medical Microbiology, Mölbis, Germany; 7 Nirvan” and “In Skin” Clinics, Vadodara, India; 8 Department of Medical Microbiology and Infectious Diseases, Canisius Wilhelmina Hospital, Nijmegen, The Netherlands; 9 Division of Mycology, Faculty of Medicine, Çukurova University, Adana, Turkey

**Keywords:** Dermatophytosis, *Trichophyton indotineae*, Outbreak, India, Multiresistance

## Abstract

A severe outbreak of highly virulent and multi-resistant dermatophytosis by species in the *Trichophyton mentagrophytes*/*T*. *interdigitale* complex is ongoing in India. The correct identity of the etiologic agent is a much-debated issue. In order to define species limits, a taxonomic study was undertaken combining molecular, morphological, and physiological characteristics as evidence of classification. Molecular characteristics show that *T. mentagrophytes s. str*. and *T. interdigitale s. str*. can be distinguished with difficulty from each other, but are unambiguously different from the Indian genotype, *T*. *indotineae* by sequences of the HMG gene. The entities were confirmed by multilocus analysis using tanglegrams. Phenotypic characters of morphology and physiology are not diagnostic, but statistically significant differences are observed between the molecular siblings. These properties may be drivers of separate evolutionary trends. *Trichophyton mentagrophytes* represents the ancestral, homothallic cloud of genotypes with a probable geophilic lifestyle, while *T*. *indotineae* and *T*. *interdigitale* behave as anthropophilic, clonal offshoots. The origin of *T*. *indotineae*, which currently causes a significant public health problem, is zoonotic, and its emergence is likely due to widespread misuse of antifungals.

**Supplementary Information:**

The online version contains supplementary material available at 10.1007/s11046-021-00544-2.

## Introduction

Dermatophytes are superficial, keratinophilic fungi which invade skin and nails causing moderate morbidity [1]. The prevalence of dermatophytosis is however very high; it has been estimated that over 20 to 25 percent of the global populations are affected [2]. Dermatophyte infections are particularly common in tropical and subtropical countries like India, where temperature and humidity are high most of the year, enhancing fungal infection [3]. The infections are generally regarded as a relatively insignificant health problem, since a wide array of effective antifungals is available. Frequently used creams contain a combination of corticosteroids and antifungal agents and suppress inflammation, leading to rapid initial improvement of the symptoms. Such medication is widely applied to patients in India to cure dermatophyte infections, usually without prescription [4]. However, in recent years, outbreaks by several dermatophyte species have been reported in southern Asia with high virulence and reduced susceptibility to commonly applied medication [5]. It has been suggested that irrational use of drug combinations may lead to resistance [6]. This may have been one of the drivers of an increased prevalence of highly virulent, multi-resistant dermatophytes at the Indian subcontinent. Other causes, such as a zoonotic origin of some of the taxa, have however not been excluded. Dermatophytosis is now rapidly emerging as a challenge for dermatologists in India [7] and spread to other continents has been observed [8–10].

*Trichophyton mentagrophytes* and *T*. *interdigitale* have been reported as being involved in the largest expansions to date [11, 12]. However, the correct identification of the Indian *Trichophyton* species is a debated issue [13, 14]. While *T. interdigitale* is regarded to be an anthropophilic species [15] mainly causing non-inflammatory tinea unguium and tinea pedis, *T*. *mentagrophytes* is thought to be zoophilic, responsible for more inflammatory dermatophytosis when infecting human hosts [16, 17]. Applying clinical criteria, the Indian strains, identified with rDNA ITS as ‘genotype VIII’ [13], should belong to *T. interdigitale* because they probably can be transmitted from human to human [14], while their relatively high virulence suggests an animal origin. Recently, Kano and coworkers [18] described a new species, *Trichophyton indotineae,* for two highly terbinafine (TBF)-resistant Indian strains, based on clinical and mycological features. Due to the limited number of strains investigated, their conclusion needs further studies to be confirmed.

Since the taxonomy of the *T*. *mentagrophytes*/*T*. *interdigitale* complex is still in dispute, with three potential names being available for the Indian outbreak, an in-depth taxonomic study is overdue. Also, the origin of the outbreak is ambiguous, while this would be essential information for public health measures. In the present study we analyzed an expanded dataset of strains from different continents, applying a combination of molecular, physiological and evolutionary parameters in order to precisely delimit species borderlines in the *T*. *mentagrophytes*/*T*. *interdigitale* species complex.

## Materials and Methods

### Strains and Culture Conditions

Reference strains were obtained from the Belgian Coordinated Collections of Microorganisms—Scientific Institute of Public Health (BCCM/IHEM, Brussels, Belgium), the Centraalbureau voor Schimmelcultures (CBS, housed at Westerdijk Fungal Biodiversity Institute, Utrecht, Netherlands), and the American Type Culture Collection (ATCC, Washington, U.S.A). Additional strains from India (*n* = 46) were provided by P. Nenoff and A. Chowdhary, and from China (*n* = 48) and Australia (*n* = 20) by P. Zhan and S. Hainsworth. We collected 50 superficial skin scrapings from patients with dermatophytosis from India, which were cultured on Taplin agar for 1–2 weeks at 28 °C. Prior to analysis, strains were cultured on Sabouraud Dextrose Agar (SDA; Oxoid, Hampshire, U.K.) for 1–2 weeks at 28 °C. The data for the 182 strains used in the study is shown in Table S-1.

### DNA Extraction and Sequencing

Mycelial fragments transferred to 1.5 mL tubes filled with 250 µL breaking buffer (2% Triton X-100, 1% SDS, 2 M NaCl, 1 M Tris–HCl pH = 8, 0.5 M EDTA pH = 8, Milli-Q water), shaken for 45 min at 70 °C at 1,400 rpm/min. Subsequently, 200 µL phenol–chloroform-isoamyl alcohol were added and shaken for 5 min at room temperature. The tubes were centrifuged for 5 min at 11,000 rpm/min. The upper layer was transferred to a new tube and stored at − 20 °C until the analysis. PCR conditions for rDNA internal transcribed spacer (ITS) and translation elongation factor 1-*α* gene (*Tef1-α*) were as follows: 95 °C for 3 min, followed by 35 cycles at 95 °C for 1 min, 50 °C for 1 min, and 72 °C for 1 min, and then an extension cycle of 72 °C for 10 min. The high-mobility group (HMG) and alpha-box gene PCR conditions were as follows: 98 °C for 3 min, followed by 40 cycles at 98 °C for 5 min, 56 °C for 5 s, and elongation at 72 °C for 45 s. The final extension cycle proceeded at 72 °C for 10 min. PCR products were visualized on 2% agarose gel. Primers are shown in Table S-2. Gel Extraction Kit (QIAquick, Hilden, Germany) was used for PCR products purification.

### Multilocus Analysis

Initial analysis of 182 strains was done with ITS. Subsequently, 113 strains representing all genotypes were selected randomly for three loci (ITS, *Tef1-α, HMG*) analyses and 29 strains were tested for the presence of alpha-box gene. Phylogenetic trees were constructed by Mega v7.0.14 using 1000 bootstrap replications with *Trichophyton benhamiae* as outgroup. Comparison of group affiliations of ITS, *Tef1-α*, and *HMG* data was performed using the R-package Dendextend.

### Genome Comparison

To further ascertain species boundaries, we compared whole-genome sequencing (WGS) data of two strains from the NCBI genome database and one strain from the literature [11], representing *T. interdigitale*, *T. mentagrophytes* and *T. indotineae*, respectively. Raw sequencing reads were quality controlled using Fastp and subsequently assembled using Spades with default parameters [19, 20]. Then, we used FastANI to compute pairwise average nucleotide identity (ANI) values among all genomes available in the NCBI database [21].

### Phenotype

*Morphology* Strains were cultured on 90-mm SDA plates, incubated at 28 °C and culture characteristics were observed after two weeks.

*Urea hydrolysis* Urea agar medium was prepared with 24 g Urea Agar Base (Oxoid, Hampshire, U.K.) in 950 mL Milli-Q water. After the medium was autoclaved and cooled to 50 °C, 50 mL of 40% Urea Solution SR0020 (Oxoid, Hampshire, UK) was added aseptically. Strains were inoculated into sterilized tubes filled with 8 mL medium and incubated at 28 °C. Color reactions were recorded after 5 days. Orange tubes and pink tubes were scored as negative and positive, respectively. Name of the fungus CBS 428.63 used as a positive control.

*Tween-80 opacity* Tween-80 agar medium was prepared according to Ates et al. [22]. Briefly, 10.0 g Bacto Peptone (Thermo Fisher Scientific, Waltham, USA), 5.0 g NaCl, 0.1 g CaCl_2_, and 15.0 g agar dissolved in 1000 mL distilled water. Five mL of autoclaved Tween-80 was added after the autoclaved medium was cooled to about 50 °C. Plates were incubated at 28 °C for two weeks. Growth was scored weekly, based on the width of the halo around the colonies to assess the lipolytic ability. A width of the halo below 2 cm is evaluated as weakly positive.

*Hair perforation* Strains were cultured in sterile Milli-Q water with blond children’s hair at 28 °C for 3 weeks and observed microscopically.

*Keratinase* Keratin azure agar was prepared according to Su et al. [23]. First, 5 mL lower layer medium (2.5% agar, 0.05% MgSO_4_·7H_2_O, 0.05% KCl, 0.05% K_2_HPO_4_, 0.01% ZnSO_4_·7H_2_O, 0.01% FeSO_4_·7H_2_O, 0.003% CuSO_4_) was prepared and sterilized into the 15 mL tubes. After the medium solidified, 0.5 mL of sterile second medium (1% agar, 0.003% CuSO_4_, 0.01% FeSO_4_·7H_2_O, 0.01% ZnSO_4_·7H_2_O, 0.05% MgSO_4_·7H_2_O, 0.05% KCl, 0.05% K_2_HPO_4_, 8 mg/ml keratin azure) was added into the same tubes as upper layer. Strains were inoculated from grown colonies on SDA plates to the tubes and incubated at 28 °C for 1 month. Release of the azure dye into the lower layer indicated keratinase production.

### Mating

In vitro mating experiments were carried on Takashio medium [3 g 2% (w/v) Sabouraud’s glucose broth (Sigma-Aldrich, St. Louis, USA), 1 g MgSO_4_·7H_2_O, 1 g KH_2_PO_4_ and 15 g agar in 1 L water], Takashio medium containing blond children’s hair, oatmeal agar (Sigma-Aldrich) and oatmeal agar containing blond children’s hair. Strains were inoculated as pairs; plates were sealed, and incubated in the dark at 30 °C for 3 months. *Arthroderma vanbreuseghemii* strains CBS 646.73 (mating type plus) and CBS 642.73 (mating type minus) were used as tester strains. The presence of sexual structures was assessed by light microscopy.

### Data Analysis

All analyses were performed using the SPSS Statistics v27.0 statistical software package (IBM, Armonk, U.S.A.). The ANOVA, Chi-square and Fisher exact tests were used to compare categorical variables between the groups. The statistical level of significance for all tests was set at 0.01.

## Results

### Genotype

The study included 182 strains in total, of which 162 strains originated from patients, 17 from animals and 3 from soil. Geographically, strains were obtained from Europe (*n* = 63), India (*n* = 46), China (*n* = 49), Japan (*n* = 2), Australia (*n* = 21) and USA. (*n* = 1). An overview of nine genotypes obtained by only ITS analyses is presented in Fig. S-1. The nearest clade to the *Trichophyton mentagrophytes*/*T*. *interdigitale* species complex, the *T*. *benhamiae* clade, was used as an outgroup. According to nomenclature proposed by Nenoff et al. [10], the ITS tree comprised *T*. *mentagrophytes* and *T*. *interdigitale* genotypes III, III*, IV, V, VII, VIII and IX (Fig. S-1), while ‘*T*. *mentagrophytes* genotype VIII’ has already been reclassified as a new species, *T*. *indotineae* by Kano et al. [18]. Strains from animal hosts (i.e., cat, dog, rabbit, and chinchilla) and soil clustered in genotypes III and III*, with the exception of four strains isolated from rabbit, cat, dog and guinea pig which clustered in *T*. *interdigitale*. Bootstrap support of the branches remained low due to the small number of mutations (Fig. S-1; Table S-1).

**Fig. 1:**
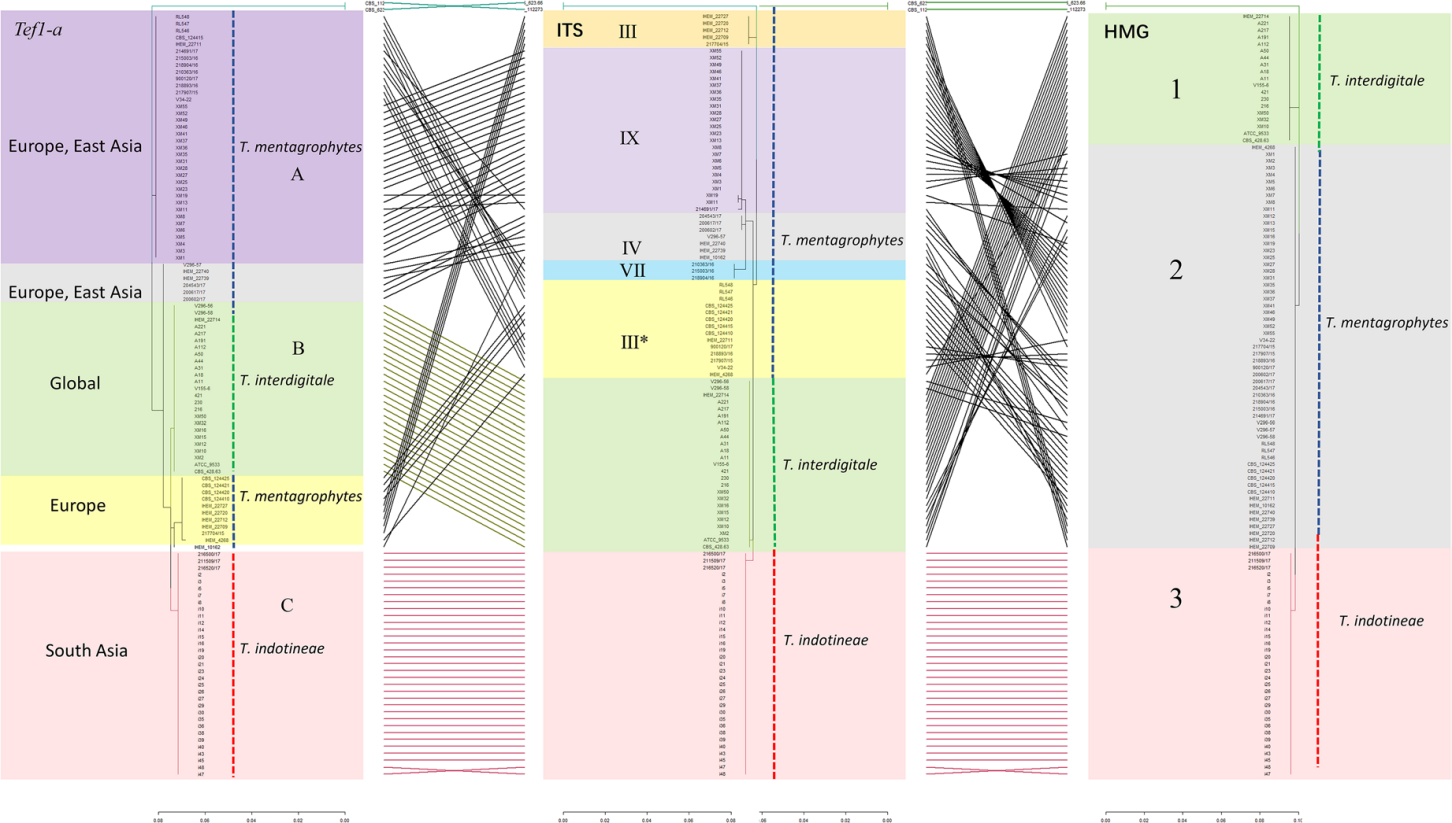
Tanglegram of 113 strains generated from *Tef1-α* (left) tree, ITS (central) tree, HMG tree (right)

**Fig. 2:**
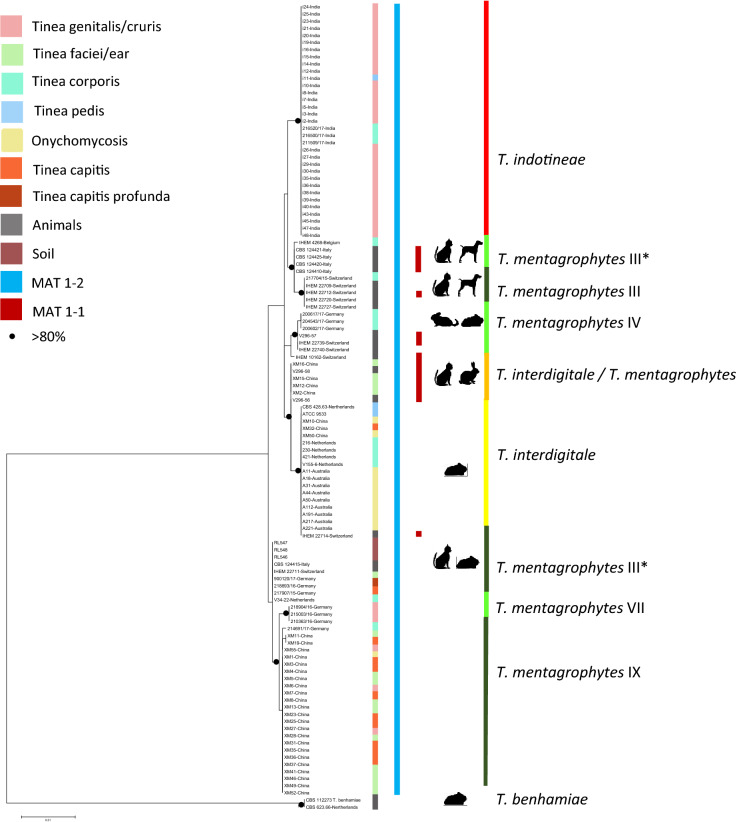
Maximum Likelihood tree generated from Tef1-α, ITS, and HMG sequences of 113 strains, combined with the source data of the strains

**Fig. 3:**
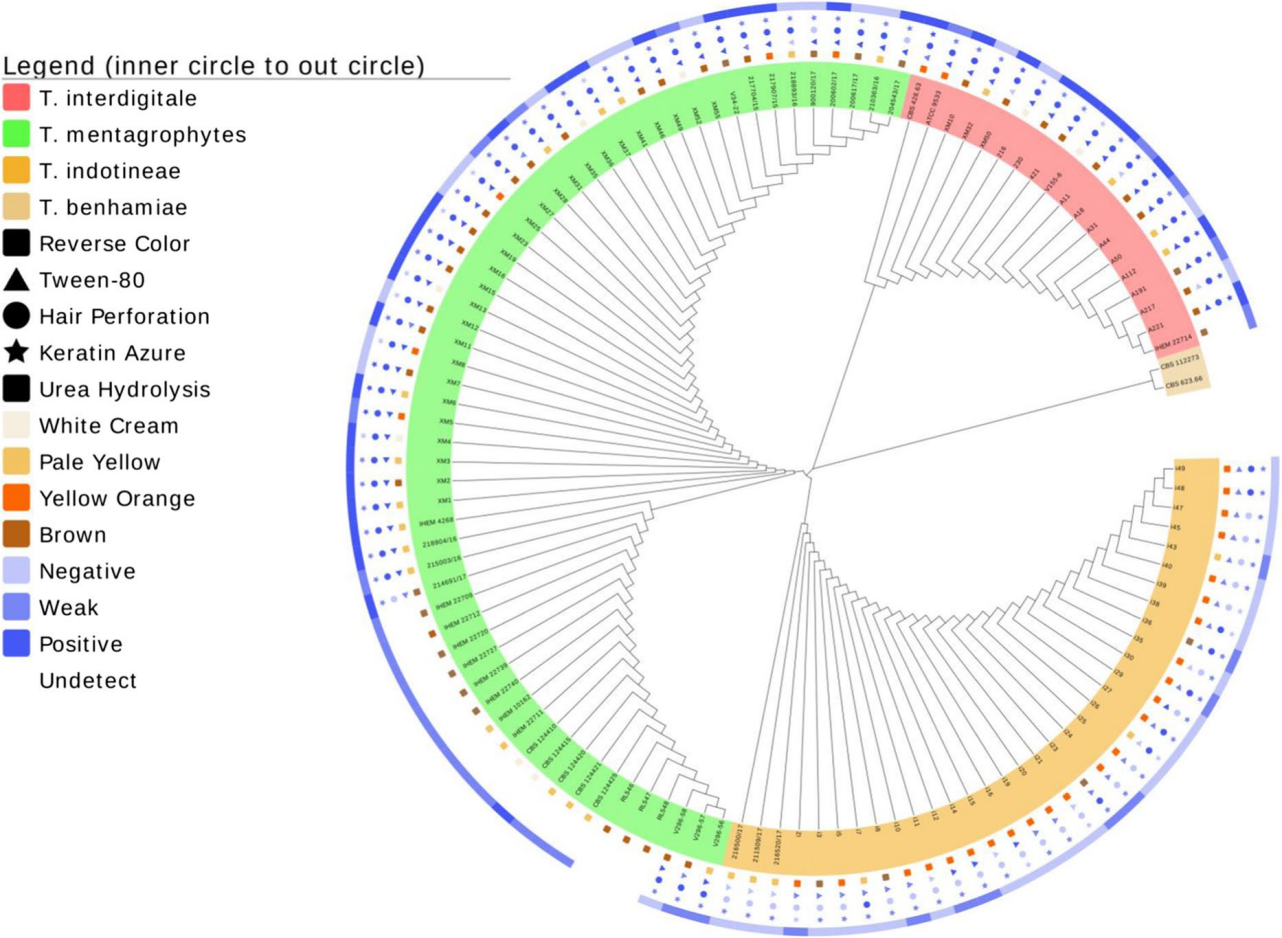
Maximum likelihood tree constructed using HMG sequences and General Time Reversible model in MEGA7; combined with morphology and physiology results *T*. *Trichophyton*

Multilocus data comparison, combining ITS, *Tef1-α*, and *HMG* loci, was performed with random selection of 113 strains representing all genotypes and additional 29 strains possessing alpha-box gene. Individual trees for each locus are shown in Fig. S-2 and Fig. 3. Comparison of multilocus trees is summarized in Fig. 1. Seventeen strains isolated from animals (*n* = 17) and humans (*n* = 4) showed both mating type genes, while the remaining strains had *HMG* mating type only (Fig. 2). The ITS locus analyses showed the highest diversity, while the mating type loci analyses showed the lowest diversity. On the basis of *Tef1-α*, six genotypes could be distinguished. Each of the *HMG* and alpha-box mating types were grouped in three genotypes, although differences within alpha-box mating type locus sequences were very small and the groups of this locus did not match with any other groups in the other analyzed genes. No alpha-box gene was found among the ITS-genotype VIII strains. Disregarding the small set of alpha-box sequences, we generated a tanglegram for the three remaining genes for 113 strains using the R-package Dendextend (Fig. 1). Red and green lines connect the congruent parts of the trees regarding the topology, while the black lines connect the incongruent parts. Two groups of *HMG* mating type, Group1 and Group 3, clustered with *T*. *interdigitale* and *T*. *indotineae*, respectively, while strains of the third Group 2 were included in *T*. *mentagrophytes* ITS genotypes III, III*, IV, VII and IX and *T*. *interdigitale* (Fig. 1). Sixteen strains of the *HMG* Group 2 (mainly *T*. *mentagrophytes*) and one strain of *HMG* Group 1 (*T*. *interdigitale*) are found homothallic, in the sense that they had both mating types in a single strain (Table S-3). In the *Tef1-α* tree, strains of *T*. *indotineae* corresponded with the same strains in the ITS tree, while the remaining strains clustered in three groups (A–C), corresponding with ITS groups III, III*, IV, VII and IX. Strain IHEM 10,162 deviates in *Tef1-α*, but clusters in *T. mentagrophytes* genotype IV with ITS.

To estimate the genetic relatedness among the genomes of the three strains, we compared the ANI value (Table 1). The FastANI values of all pairs exceeded 95%, which is generally is taken to indicate conspecificity [21].

**Table 1:** Whole genome sequencing FastANI value of three strains

Nomenclature	Number	FastANI value (%)
*T*. *mentagrophytes* vs. *T*. *interdigitale*	D15P127 vs. H6	98.7
*T*. *mentagrophytes* vs. *T*. *indotineae*	D15P127 vs. P15-822	97.42
*T*. *interdigitale* vs. *T*. *indotineae*	H6 vs. P15-822	98.75

Based on these results, it is suggested that the three species under discussion are optimally distinguished with *HMG* locus analyses as three main genotypic groups containing the type strains of *T*. *indotineae* (CBS 146623), *T*. *interdigitale* (CBS 428.63), and *T*. *mentagrophytes* (IHEM 4268), and having approximate differences in geographic distribution (Fig. 1). Note that in multilocus comparison (Fig. 2), *T*. *mentagrophytes* Genotype III* disintegrates in two separate groups. *Trichophyton indotineae* and *T. interdigitale* show low diversity, with only a single genotype in all three genes, while *T. mentagrophytes* the highest, having six genotypes with ITS and five (A–C plus IHEM 10162) with *Tef1-α*. Applying these specific circumscriptions, strains of animal origin and from soil all clustered in *T. mentagrophytes* ITS genotypes III, with three animal strains clustering in *T. interdigitale* (Fig. 3).

### Phenotype

Strains belonging to *Trichophyton indotineae, T*. *mentagrophytes* and *T*. *interdigitale* as defined above all have expanding, cottony to powdery colonies. The colony reverse shows cream colored, yellow–orange, pale brown, brown ochre or yellow–brown pigmentation (Fig. 3). The reverse of *T*. *interdigitale* strains was mostly brown ochre, that of *T*. *mentagrophytes* mostly brown ochre to pale brown, and *T*. *indotineae* mostly pale brown to yellow–orange. Chi-square and Fisher tests show that the differences between species are significant (*χ*^2^ = 69.648, *p* < 0.01). Yellow pigmented strains, including the type strain of *T*. *interdigitale* var. *nodulare* CBS 429.63, often show reduced sporulation and formation of chlamydospore-like cellular clumps.

**Fig. 4:**
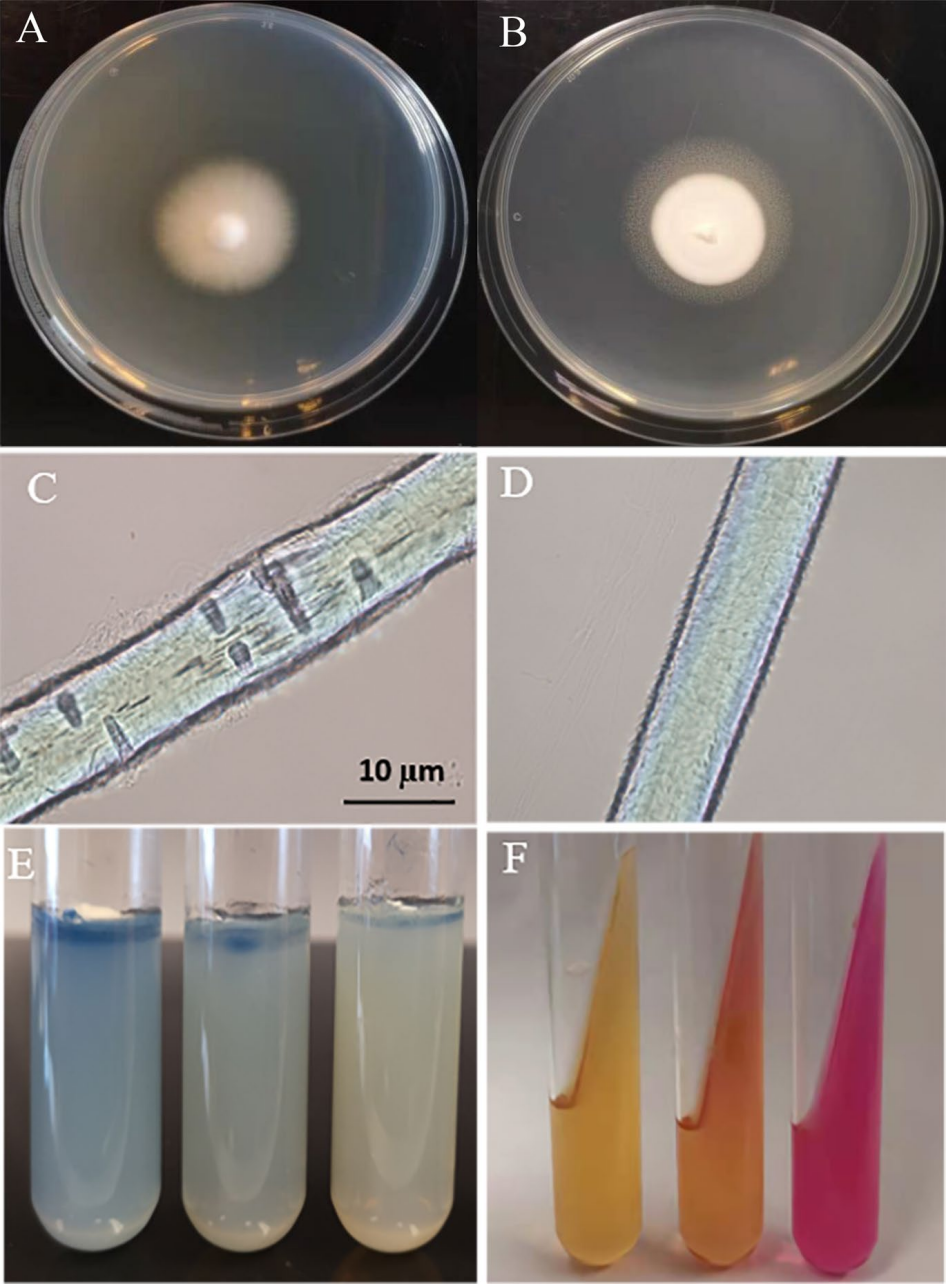
Phenotypic methodology followed in the study. **a** Tween-80 opacity negative result **b** Tween-80 opacity positive result. **c** Hair perforation test positive result. **d** Hair perforation test negative result **e** Keratin azure agar evaluation from left to right; positive, weakly positive, negative. **f** Evaluation of urease activity; from left to right; negative, weakly positive and positive

Results of physiological tests are presented in Fig. 3, Fig. 4 and Table S-4. The Tween-80 opacity test was conducted to verify lipolytic abilities of the dermatophytes. All strains of *T*. *interdigitale*, 76% of *T*. *indotineae*, and 95% of *T*. *mentagrophytes* showed the positive or weakly positive results. No significant difference was found between *T*. *interdigitale* and *T*. *mentagrophytes* (*p* > 0.05) for lipolytic activity, whereas their difference between *T*. *indotineae* was found significant (*p* < 0.01).

All strains of *T. interdigitale* revealed positive result with hair perforation test, as well as 92.5% of *T. mentagrophytes* and 27.08% of *T*. *indotineae* strains. The difference between *T*. *interdigitale* and *T*. *mentagrophytes* strains was not statistically significant (*p* > 0.05), whereas the difference between these two species and *T*. *indotineae* was found significant (*p* < 0.01).

The majority of the strains showed positive or weakly positive result with the keratin azure test, especially for *T*. *indotineae* (85.1%) and *T*. *mentagrophytes* (68.51%), compared to *T*. *interdigitale* (95.24%). The difference between *T*. *interdigitale* on the one hand and *T*. *mentagrophytes* and *T*. *indotineae* on the other was statistically significant (*p* < 0.01), whereas the difference between *T*. *mentagrophytes* and *T*. *indotineae* was not significant (*p* > 0.05).

Results of urea hydrolysis tests are shown in Figs. 3 and 4 and Table S-4. Positive and weakly positive urea hydrolysis were observed in 75%, 67.2% and 27.1% of the strains in *T*. *interdigitale*, *T*. *mentagrophytes* and *T*. *indotineae*, respectively. There was no statistically significant difference in urea hydrolysis between *T*. *interdigitale* and *T*. *mentagrophytes* (*p* > 0.05), whereas their difference with *T*. *indotineae* was found significant (*p* < 0.01).

### Mating

Twenty-two strains were randomly selected from different species as two groups; one carrying the alpha-box gene and another group carrying the *HMG* gene. Two animal strains had both *HMG* and alpha-box genes. The tester strains *A. vanbreuseghemii* CBS 646.73 (RV27960) and CBS 642.73 (RV27961) were found successfully mated in vitro by Hejtmánek [24]; however, in the current study they had lost the ability to produce gymnothecia. Only a single pair of *T*. *interdigitale* strains, Number 335 and CBS 428.63, were able to produce gymnothecia on Takashio agar with hair at 30 °C, but no ascospores were found with microscopy.

### Clinical Appearance

The origins of the strains are shown in Fig. 2. *Trichophyton interdigitale* was prevalently isolated from superficial infections on exposed body sites such as scalp and face, while also feet and nails were frequently infected with this species. *Trichophyton mentagrophytes* has a similar predilection but is also often found on trunk and genitals. *Trichophyton indotineae* is mostly restricted to trunk and groin. The difference in clinical distributions of the three species was found significant (*p* < 0.01).

## Discussion

The aim of the current study is to resolve the taxonomy of the *Trichophyton mentagrophytes*/*T*. *interdigitale* complex using a polyphasic approach. Nenoff et al. [10] and Heidemann et al. [25] distinguished nine genotypes in the complex on the basis of rDNA ITS and the partial *Tef1-α* gene. Their ‘genotype VIII’ is a clone that emerges in India and is characterized by elevated virulence and frequent occurrence of terbinafine (TBF) resistance. Appropriate naming of the emerging clone in India is essential, but on the basis of the available data it could not be unambiguously identified as either *T*. *mentagrophytes* or *T*. *interdigitale* [11]. On the basis of the ITS data, Kano et al. [18] described a new species, *T*. *indotineae*, for two strains originating from Indian patients. These strains proved to be molecularly identical to ‘genotype VIII’. Morphological and physiological differences between *T*. *mentagrophytes* and *T. interdigitale* are small compared to the high ITS diversity, with nine genotypes in total [11]. Other genetic markers such as *β*-tubulin are also used for multilocus phylogeny, but in anthropophilic dermatophytes only few genes provide sufficient evidence for stable classification [26]. In our multilocus approach, we therefore combined ITS and *Tef1-α* with mating type genes *HMG* and *α*-box, which seem to be drivers of evolution in several dermatophyte groups [27]. The *HMG* gene, which is preponderant in the *T*. *mentagrophytes* complex (in contrast to *T*. *rubrum* where the *α*-box is nearly exclusively present), provided a clear-cut grouping of two entities matching with the genotypes *T*. *mentagrophytes* and *T*. *indotineae. Trichophyton interdigitale* could not be separated unambiguously, as six strains showed multilocus conflict. Two of these strains were isolated from animals and all six strains were found homothallic. The topologies of ITS and *Tef1-α* trees were congruent, with a higher level of diversity in ITS locus. However, the whole genomes of *T. mentagrophytes*, *T. interdigitale* and *T. indotineae* are very similar (FastANI values > 95%). Additionally, ITS genotypes III, III*, IV, VII and IX all corresponded with a single *HMG* genotype. No *α*-box gene was detected in any of the *T*. *indotineae* strains, suggesting a clonal outbreak population structure for this species.

Differences between groups in anonymous markers need to be supported by phenotypic characteristics, as these are the evolutionary drivers of segregation and adaptation. Macromorphologically, *T*. *interdigitale*, *T*. *mentagrophytes* and *T*. *indotineae* just differ slightly in the pigmentation of the colony reverse. Most colonies of *T*. *interdigitale* are brown ochre, except for the occasional strains of yellow var. *nodulare* which can be regarded as a mutant; those of *T. mentagrophytes* are brown ochre to pale brown, and *T*. *indotineae* colonies had pale brown to yellow orange pigmentation. While *T*. *interdigitale* and *T*. *indotineae* differed significantly (*p* < 0.01), *T*. *mentagrophytes* took an intermediate position. Physiologically, *T*. *interdigitale* and *T*. *mentagrophytes* are similar, and different from *T*. *indotineae*. Urea hydrolysis was found mostly positive in *T*. *interdigitale* and *T*. *mentagrophytes*, while most strains of *T*. *indotineae* strains were weakly positive or negative.

Lipolytic activity differs considerably between dermatophyte species [28]. For example, *T*. *rubrum* does not show lipolysis, whereas *T*. *mentagrophytes* does. In our study, the lipolytic abilities of *T*. *mentagrophytes* and *T*. *interdigitale* were very similar, and were higher than those of *T*. *indotineae*. Possibly this is associated with a higher prevalence of *T*. *mentagrophytes* on the human scalp, which is relatively rich in lipids [28]. However, we noted that one of the strains of *T*. *mentagrophytes* isolated from tinea capitis profunda did not have lipolytic ability. Ates et al. [22] observed that in vitro hair perforation in *T*. *mentagrophytes* and *T*. *interdigitale* matched with their lipolytic ability. In the current study, a significantly higher ability of hair perforation was observed in *T*. *interdigitale* and *T*. *mentagrophytes* compared to *T*. *indotineae* (*p* < 0.01). Keratin degradation was significantly larger in *T*. *interdigitale* than in *T*. *mentagrophytes* and *T*. *indotineae* (*p* < 0.01). This might be linked to the prevalent habitat of *T*. *indotineae* in tinea corporis/tinea cruris and *T. mentagrophytes* in tinea faciei/tinea capitis, while *T*. *interdigitale* strains were frequently isolated from tinea pedis and tinea unguium. The slightly different clinical predilections of the species may enhance the differential specialization in the course of evolution.

*Trichophyton mentagrophytes* has long been regarded as a zoophilic species [29]. However, the species is also commonly found on humans (Fig. 2). Several studies were conducted to distinguish zoophilic and anthropophilic strains of *T*. *mentagrophytes* [10]. However, the distinction is problematic, as the source of human infections mostly cannot be traced. For our study, 17 strains originated from different animals, and three isolates were obtained from soil. Nearly all of these isolates were located in *T*. *mentagrophytes* genotype III (Fig. 2), while three strains from animals belonged to *T*. *interdigitale*; none of the animal isolates was found in *T*. *indotineae*. Berlin et al. [30] also reported *T*. *interdigitale* from guinea pigs and hypothesized that these strains had a human origin from the breeders.

The mating type distribution in the *T*. *mentagrophytes*/*T*. *interdigitale* complex is highly unbalanced, with *HMG vs*. *α*-box ratio being 113:29. Symoens et al. [31] noted the production of sterile gymnothecia in *T*. *interdigitale*; also in our study, no ascospores were produced, despite several attempts with several different mating conditions. This may indicate the loss of sexuality, as is commonly observed in anthropophilic species [31]. In the current study, *T*. *mentagrophytes* is found as the most variable entity in the complex, suggesting ancestry. Genotype III of this species contains nearly all animal and soil isolates. Strains with both mating types *HMG* and *α*-box are also mainly located in *T*. *mentagrophytes.* This is consistent with the hypothesis suggesting that *T*. *mentagrophytes* originally was a geophilic or zoophilic, sexual species, from which ‘clonal offshoots’ *T*. *interdigitale* and *T*. *indotineae* emerged [32]; genotype III seems closest to the species’ original condition.

**Fig. 5:**
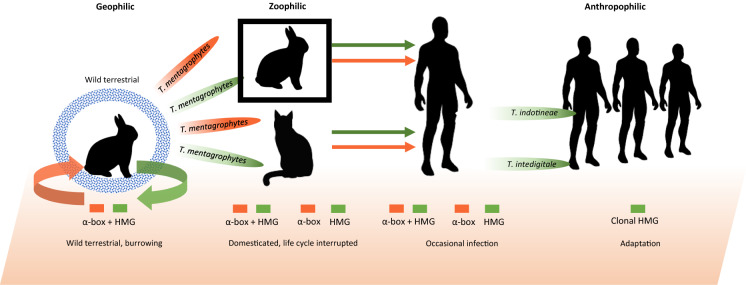
Summary of the hypothesis proposed in the current study. Terrestrial ancestral species harboring both mating types and able to infect burrowing animals is producing populations which are clonal and highly adapted to the human host

In conclusion, it seems likely that *Trichophyton mentagrophytes*, represented by genotypes III, III* and IV, originally was a geophilic species, and is going through a process of adaptation to the human host releasing several clonal populations with higher degrees of human adaptation (Fig. 5). Soilborne strains are likely to occur with terrestrial, burrowing wild animals, such as rabbits. Homothallism (i.e. both mating types present in a single strain) is relatively frequent among the animal strains, suggesting a complete life cycle with fertile gymnothecia. Rabbits are also maintained in captivity. Under the conditions of domestication, with novel hosts like guinea pig, chinchilla, cat and dog, the soilborne part of the life cycle is interrupted, and propagation becomes predominantly clonal. Several authors have reported human infections among rabbit breeders [33–35]. Host switch from animals to humans is a next possible evolutionary step. The most recent, Indian clone, *T*. *indotineae*, seems quite successful for this kind of adaptation; *T*. *interdigitale* may have gone through this process in an earlier stage, since it still contains strains carrying both mating types and is not entirely separate from *T*. *mentagrophytes*. According to this hypothesis, we witness an evolution from a geophilic, sexual life style in wild animals, via zoophily in domesticated animals, to clonal expansion on humans in successful anthropophily, within a single species complex. Still, this hypothesis would need more strains from burrows of wild animals to be confirmed. The offshoots are genetically still very close to the ancestral core. A main reason to use species names for these entities is their epidemic behavior on humans; if limited to non-human habitats, the genotypes probably would not have remarkable. The attribution of names to these dermatophytes thus has a practical rather than a scientific justification.

A rather dramatic and rapid epidemiological shift from prevalence of *T. rubrum* to *T. mentagrophytes* has been observed in the epidemic-like scenario of dermatophytosis in India [34]. It fortuitously coincides with free availability of topical corticosteroid creams and, more importantly, with growing availability and sales of hazardous fixed dose combination creams (FDCs). The majority of these contain the super potent topical steroid clobetasol propionate mixed with miconazole/clotrimazole/terbinafine and antibacterial agents like neomycin/gentamycin/ofloxacin [4]. They are sold over the counter and are rampantly abused, often for months or years [4, 34]. They lead to steroid-induced suppression of local cellular immunity as well as an altered cutaneous microbiome providing a window of opportunity for the unique, multidrug-resistant species *Trichophyton indotineae* (Genotype VIII) [10, 18]. The export of these creams to several countries and their use by migrants and travellers from the Indian subcontinent, pose a significant global threat in the form of a large pool of inadequately treated infectious patients propagating the disease [10]. Essential public health measures include ensuring sale of topical steroids and their combinations by prescription only, disallowing permissions to manufacture irrational and hazardous combination creams (FDCs), which all need strong and continuing advocacy of rational therapy of dermatophytoses by dermatologists and public health professionals [34].

## Supplementary Information

Below is the link to the electronic supplementary material.

Supplementary file1 (PDF 98 kb)

Supplementary file2 (PDF 80 kb)

Supplementary file3 (DOCX 39 kb)

Supplementary file4 (DOCX 19 kb)

Supplementary file5 (DOCX 16 kb)

Supplementary file6 (DOCX 16 kb)
