# Supplementary figures and images for: Taxonomy of the Trichophyton mentagrophytes/T. interdigitale Species Complex Harboring the Highly Virulent, Multiresistant Genotype T. indotineae
Source: Mycopathologia. 2021 Apr 13;186(3):315–26. doi: 10.1007/s11046-021-00544-2 (PMC8249266; doi:10.1007/s11046-021-00544-2)

Fig. S-1 ITS tree

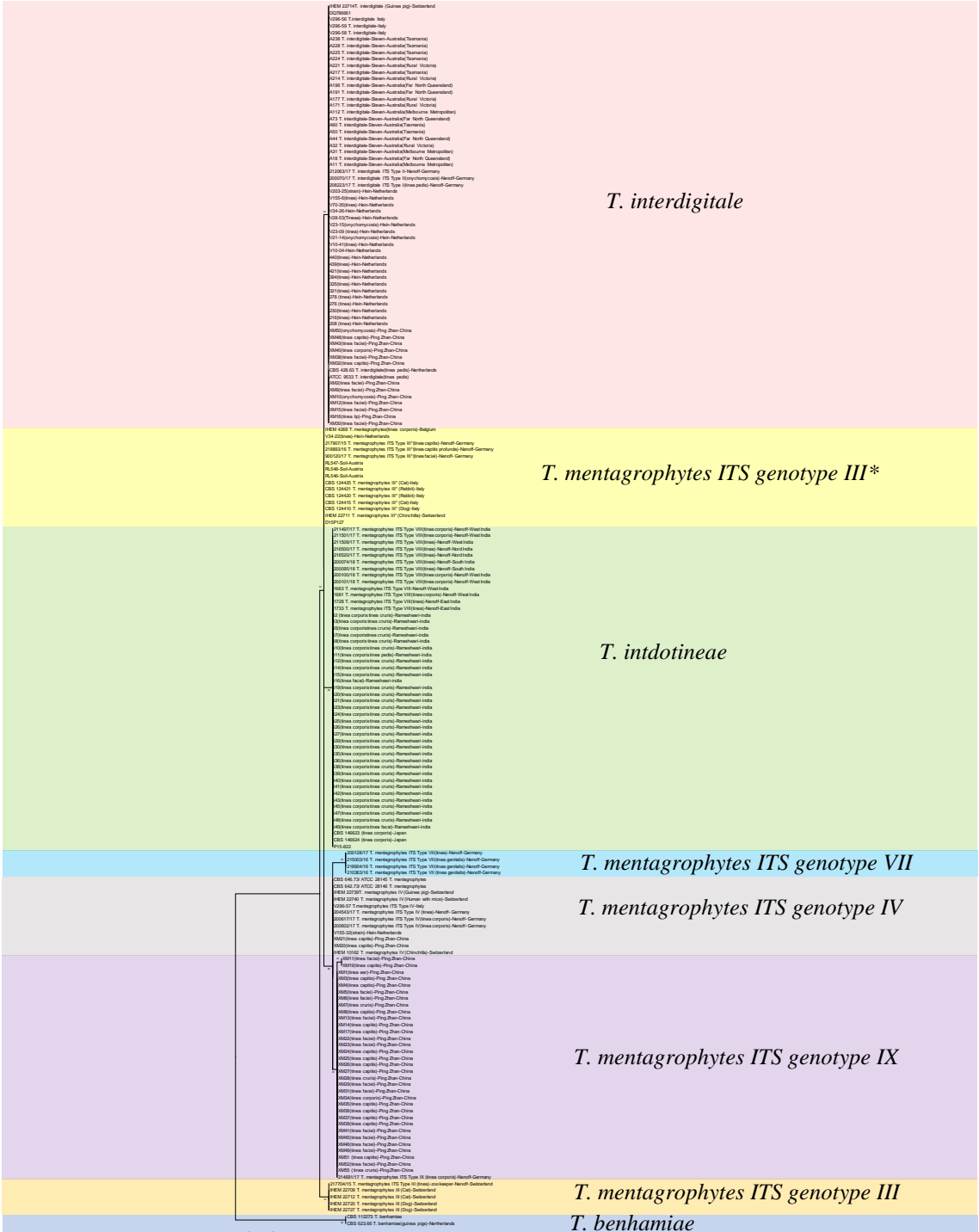

Supplement: Supplementary file 1 — Supplementary file1 (PDF 98 kb) [file 11046_2021_544_MOESM1_ESM.pdf]

Fig S-2

*Tef1-a*

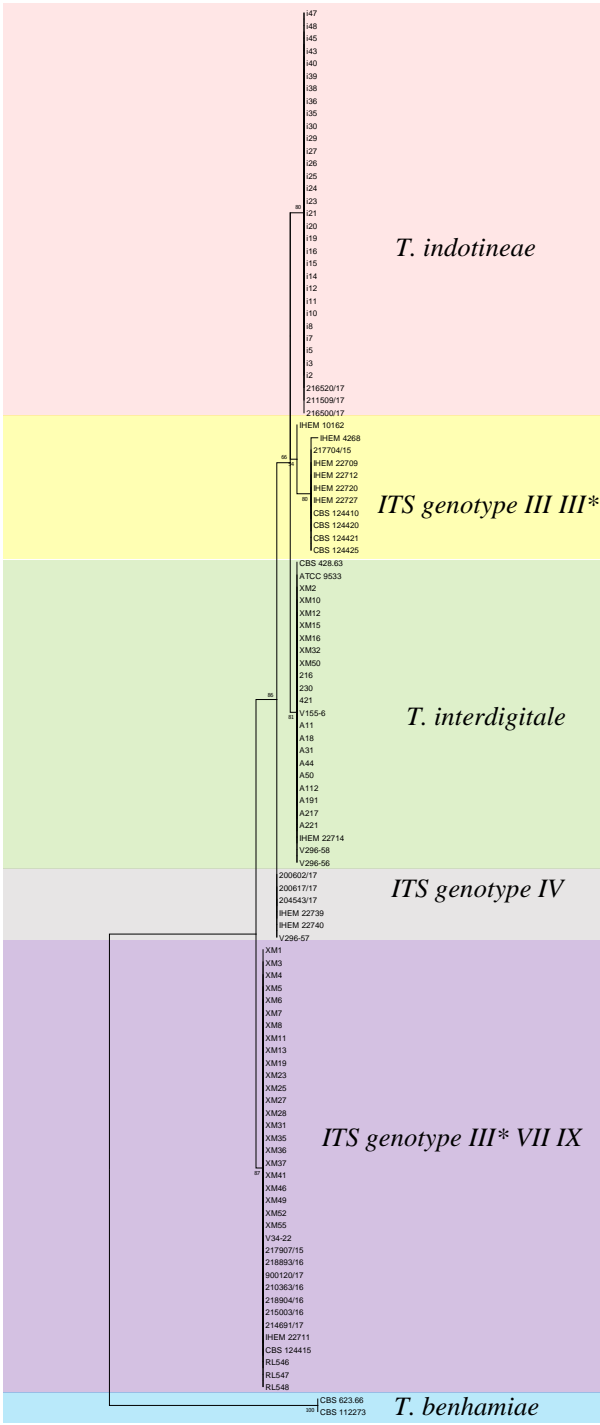

*ITS*

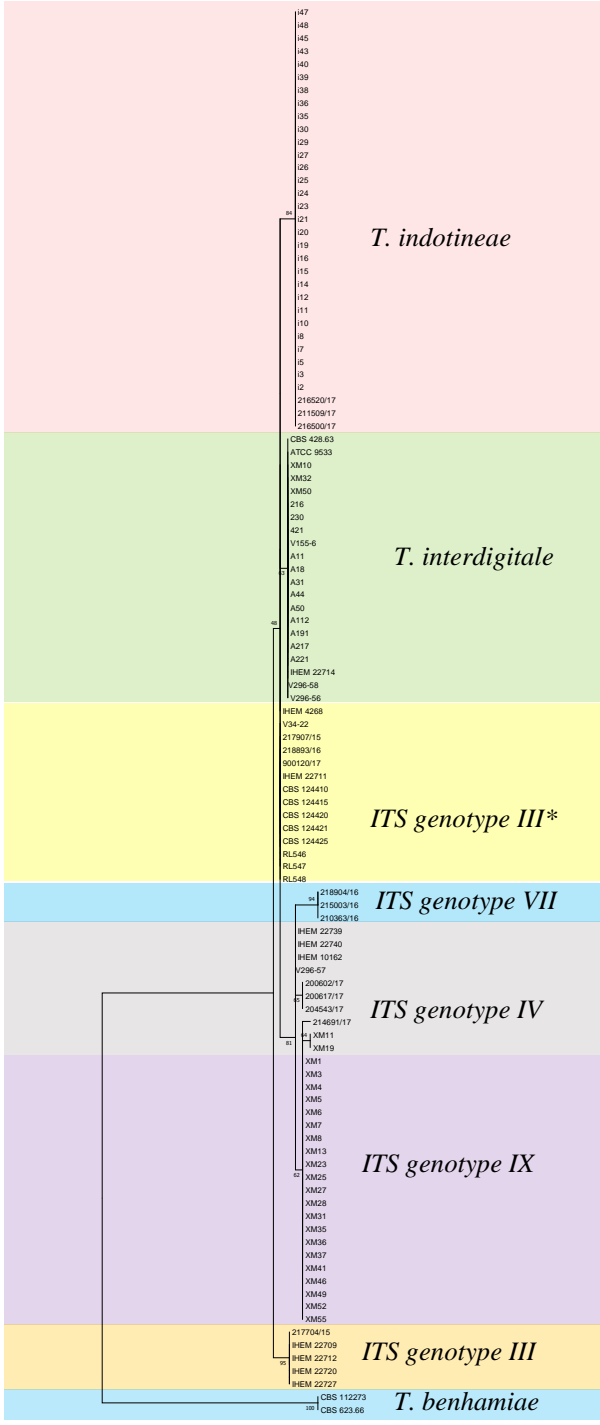

*Alpha-box*

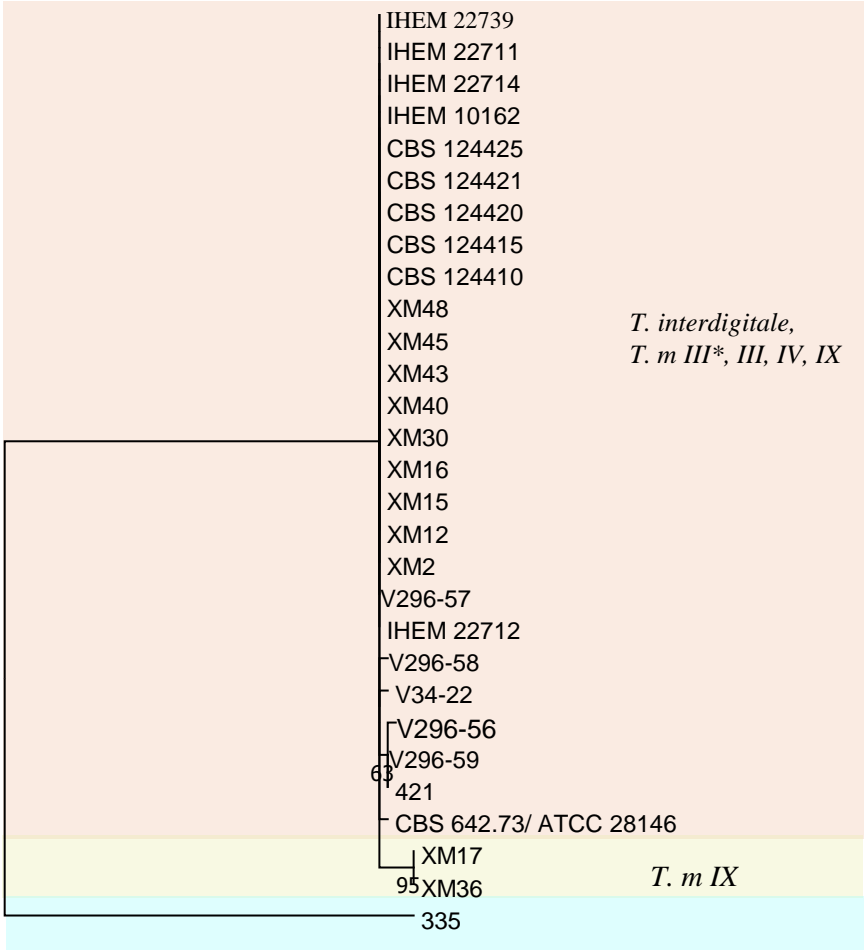

Supplement: Supplementary file 2 — Supplementary file2 (PDF 80 kb) [file 11046_2021_544_MOESM2_ESM.pdf]
