# Supplementary material for: Taxonomy of the Trichophyton mentagrophytes/T. interdigitale Species Complex Harboring the Highly Virulent, Multiresistant Genotype T. indotineae
Source: Mycopathologia. 2021 Apr 13;186(3):315–26. doi: 10.1007/s11046-021-00544-2 (PMC8249266; doi:10.1007/s11046-021-00544-2)
Supplement: Supplementary file 3 — Supplementary file3 (DOCX 39 kb) [file 11046_2021_544_MOESM3_ESM.docx]

**Table S-1.** Information of Strains

| Group | Number | Country | Source | Collect by | ITS Genotype |
| --- | --- | --- | --- | --- | --- |
| A | IHEM 22714 | Switzerland | Guinea pig | Michel Monod | T. interdigitale |
| A | XM2 | China | Tinea faciei | Ping Zhan | T. interdigitale |
| A | XM9 | China | Tinea faciei | Ping Zhan | T. interdigitale |
| A | XM10 | China | Onychomycosis | Ping Zhan | T. interdigitale |
| A | XM12 | China | Tinea faciei | Ping Zhan | T. interdigitale |
| A | XM15 | China | Tinea faciei | Ping Zhan | T. interdigitale |
| A | XM16 | China | Tinea faciei | Ping Zhan | T. interdigitale |
| A | XM30 | China | Tinea faciei | Ping Zhan | T. interdigitale |
| A | XM32 | China | Tinea capitis | Ping Zhan | T. interdigitale |
| A | XM40 | China | Tinea corporis | Ping Zhan | T. interdigitale |
| A | XM43 | China | Tinea faciei | Ping Zhan | T. interdigitale |
| A | XM48 | China | Tinea capitis | Ping Zhan | T. interdigitale |
| A | XM50 | China | Onychomycosis | Ping Zhan | T. interdigitale |
| A | 208 | Netherlands | Tinea | Hein van der Lee | T. interdigitale |
| A | 216 | Netherlands | Tinea | Hein van der Lee | T. interdigitale |
| A | 230 | Netherlands | Tinea | Hein van der Lee | T. interdigitale |
| A | 276 | Netherlands | Tinea | Hein van der Lee | T. interdigitale |
| A | 278 | Netherlands | Tinea | Hein van der Lee | T. interdigitale |
| A | 321 | Netherlands | Tinea | Hein van der Lee | T. interdigitale |
| A | 335 | Netherlands | Tinea | Hein van der Lee | T. interdigitale |
| A | 394 | Netherlands | Tinea | Hein van der Lee | T. interdigitale |
| A | 421 | Netherlands | Tinea | Hein van der Lee | T. interdigitale |
| A | 439 | Netherlands | Tinea | Hein van der Lee | T. interdigitale |
| A | 440 | Netherlands | Tinea | Hein van der Lee | T. interdigitale |
| A | V10-04 | Netherlands | unknown | Hein van der Lee | T. interdigitale |
| A | V10-41 | Netherlands | Tinea | Hein van der Lee | T. interdigitale |
| A | V21-14 | Netherlands | Onychomycosis | Hein van der Lee | T. interdigitale |
| A | V23-09 | Netherlands | Tinea | Hein van der Lee | T. interdigitale |
| A | V23-15 | Netherlands | Onychomycosis | Hein van der Lee | T. interdigitale |
| A | V28-53 | Netherlands | Tinea | Hein van der Lee | T. interdigitale |
| A | V34-26 | Netherlands | unknown | Hein van der Lee | T. interdigitale |
| A | V70-35 | Netherlands | Tinea | Hein van der Lee | T. interdigitale |
| A | V155-6 | Netherlands | unknown | Hein van der Lee | T. interdigitale |
| A | V203-25 | Netherlands | unknown | Hein van der Lee | T. interdigitale |
| A | ATCC 9533 | U.S.A. | Tinea pedis | unknown | T. interdigitale |
| A | 208223/17 | Germany | Tinea pedis | Pietro Nenoff | T. interdigitale |
| A | A11 | Australia | Onychomycosis | Steven Hainsworth | T. interdigitale |
| A | A18 | Australia | Onychomycosis | Steven Hainsworth | T. interdigitale |
| A | A31 | Australia | Onychomycosis | Steven Hainsworth | T. interdigitale |
| A | A32 | Australia | Onychomycosis | Steven Hainsworth | T. interdigitale |
| A | A44 | Australia | Onychomycosis | Steven Hainsworth | T. interdigitale |
| A | A50 | Australia | Onychomycosis | Steven Hainsworth | T. interdigitale |
| A | A60 | Australia | Onychomycosis | Steven Hainsworth | T. interdigitale |
| A | A73 | Australia | Onychomycosis | Steven Hainsworth | T. interdigitale |
| A | A112 | Australia | Onychomycosis | Steven Hainsworth | T. interdigitale |
| A | A171 | Australia | Onychomycosis | Steven Hainsworth | T. interdigitale |
| A | A177 | Australia | Onychomycosis | Steven Hainsworth | T. interdigitale |
| A | A191 | Australia | Onychomycosis | Steven Hainsworth | T. interdigitale |
| A | A196 | Australia | Onychomycosis | Steven Hainsworth | T. interdigitale |
| A | A214 | Australia | Onychomycosis | Steven Hainsworth | T. interdigitale |
| A | A217 | Australia | Onychomycosis | Steven Hainsworth | T. interdigitale |
| A | A221 | Australia | Onychomycosis | Steven Hainsworth | T. interdigitale |
| A | A224 | Australia | Onychomycosis | Steven Hainsworth | T. interdigitale |
| A | A225 | Australia | Onychomycosis | Steven Hainsworth | T. interdigitale |
| A | A228 | Australia | Onychomycosis | Steven Hainsworth | T. interdigitale |
| A | A238 | Australia | Onychomycosis | Steven Hainsworth | T. interdigitale |
| A | V296-56 | Italy | Cat | Simona Nardoni | T. interdigitale |
| A | V296-59 | Italy | Dog | Simona Nardoni | T. interdigitale |
| A | CBS 428.63 | Netherlands | Tinea pedis | unknown | T. interdigitale |
| A | V296-58 | Italy | Rabbit | Simona Nardoni | T. interdigitale |
| A | XM38 | China | Tinea faciei | Ping Zhan | T. interdigitale |
| A | 212063/17 | Germany | unknown | Pietro Nenoff | T. interdigitale |
| A | 200070/17 | Germany | Onychomycosis | Pietro Nenoff | T. interdigitale |
| B | CBS 124425 | Italy | Cat | unknown | T. mentagrophytes III* |
| B | CBS 124421 | Italy | Rabbit | unknown | T. mentagrophytes III* |
| B | CBS 124420 | Italy | Rabbit | unknown | T. mentagrophytes III* |
| B | CBS 124415 | Italy | Cat | unknown | T. mentagrophytes III* |
| B | CBS 124410 | Italy | Dog | unknown | T. mentagrophytes III* |
| B | RL546 | Slovakia | Soil | Roman Labuda | T. mentagrophytes III* |
| B | RL547 | Slovakia | Soil | Roman Labuda | T. mentagrophytes III* |
| B | RL548 | Slovakia | Soil | Roman Labuda | T. mentagrophytes III* |
| B | IHEM 22711 | Switzerland | Dog | Michel Monod | T. mentagrophytes III* |
| B | V34-22 | Netherlands | Tinea | Hein van der Lee | T. interdigitale |
| B | V218-10 | Netherlands | unknown | Hein van der Lee | T. mentagrophytes III* |
| B | V21-63 | Netherlands | Tinea | Hein van der Lee | T. mentagrophytes III* |
| B | IHEM 4268 | Belgium | Tinea corporis | Michel Monod | T. mentagrophytes III* |
| B | 217907/15 | Germany | Tinea capitis | Pietro Nenoff | T. mentagrophytes III* |
| B | 218893/16 | Germany | Tinea capitis profunda | Pietro Nenoff | T. mentagrophytes III* |
| B | 900120/17 | Germany | Tinea faciei | Pietro Nenoff | T. mentagrophytes III* |
| B | IHEM 22709 | Switzerland | Cat | Michel Monod | T. mentagrophytes III |
| B | IHEM 22712 | Switzerland | Cat | Michel Monod | T. mentagrophytes III |
| B | IHEM 22720 | Switzerland | Dog | Michel Monod | T. mentagrophytes III |
| B | IHEM 22727 | Switzerland | Dog | Michel Monod | T. mentagrophytes III |
| B | 217704/15 | Switzerland | (tinea)-zoo keeper | Pietro Nenoff | T. mentagrophytes III |
| B | IHEM 22739 | Switzerland | Mouse | Michel Monod | T. mentagrophytes IV |
| B | IHEM 22740 | Switzerland | Human with mice | Michel Monod | T. mentagrophytes IV |
| B | IHEM 10162 | Switzerland | Chinchilla | Michel Monod | T. mentagrophytes IV |
| B | 200602/17 | Germany | Tinea corporis | Pietro Nenoff | T. mentagrophytes IV |
| B | V296-57 | Italy | Chinchilla | Simona Nardoni | T. mentagrophytes IV |
| B | CBS 646.73 | Netherlands | unknown | unknown | T. mentagrophytes IV |
| B | CBS 642.73 | Netherlands | unknown | unknown | T. mentagrophytes IV |
| B | 200617/17 | Germany | Tinea corporis | Pietro Nenoff | T. mentagrophytes IV |
| B | 204543/17 | Germany | Tinea | Pietro Nenoff | T. mentagrophytes IV |
| B | V155-32 | Netherlands | unknown | Hein van der Lee | T. mentagrophytes IV |
| B | XM20 | China | Tinea capitis | Ping Zhan | T. mentagrophytes IV |
| B | XM21 | China | Tinea capitis | Ping Zhan | T. mentagrophytes IV |
| B | 210363/16 | Germany | Tinea genitalis | Pietro Nenoff | T. mentagrophytes VII |
| B | 218904/16 | Germany | Tinea genitalis | Pietro Nenoff | T. mentagrophytes VII |
| B | 215003/16 | Germany | Tinea genitalis | Pietro Nenoff | T. mentagrophytes VII |
| B | 200128/17 | Germany | Tinea | Pietro Nenoff | T. mentagrophytes VII |
| B | XM1 | China | Tinea ear | Ping Zhan | T. mentagrophytes IX |
| B | XM3 | China | Tinea capitis | Ping Zhan | T. mentagrophytes IX |
| B | XM4 | China | Tinea capitis | Ping Zhan | T. mentagrophytes IX |
| B | XM5 | China | Tinea faciei | Ping Zhan | T. mentagrophytes IX |
| B | XM6 | China | Tinea faciei | Ping Zhan | T. mentagrophytes IX |
| B | XM7 | China | Tinea cruris | Ping Zhan | T. mentagrophytes IX |
| B | XM8 | China | Tinea capitis | Ping Zhan | T. mentagrophytes IX |
| B | XM11 | China | Tinea faciei | Ping Zhan | T. mentagrophytes IX |
| B | XM13 | China | Tinea faciei | Ping Zhan | T. mentagrophytes IX |
| B | XM14 | China | Tinea capitis | Ping Zhan | T. mentagrophytes IX |
| B | XM17 | China | Tinea capitis | Ping Zhan | T. mentagrophytes IX |
| B | XM19 | China | Tinea capitis | Ping Zhan | T. mentagrophytes IX |
| B | XM22 | China | Tinea faciei | Ping Zhan | T. mentagrophytes IX |
| B | XM23 | China | Tinea faciei | Ping Zhan | T. mentagrophytes IX |
| B | XM24 | China | Tinea capitis | Ping Zhan | T. mentagrophytes IX |
| B | XM25 | China | Tinea capitis | Ping Zhan | T. mentagrophytes IX |
| B | XM26 | China | Tinea capitis | Ping Zhan | T. mentagrophytes IX |
| B | XM27 | China | Tinea capitis | Ping Zhan | T. mentagrophytes IX |
| B | XM28 | China | Tinea cruris | Ping Zhan | T. mentagrophytes IX |
| B | XM29 | China | Tinea faciei | Ping Zhan | T. mentagrophytes IX |
| B | XM31 | China | Tinea faciei | Ping Zhan | T. mentagrophytes IX |
| B | XM34 | China | Tinea corporis | Ping Zhan | T. mentagrophytes IX |
| B | XM35 | China | Tinea capitis | Ping Zhan | T. mentagrophytes IX |
| B | XM36 | China | Tinea capitis | Ping Zhan | T. mentagrophytes IX |
| B | XM37 | China | Tinea capitis | Ping Zhan | T. mentagrophytes IX |
| B | XM39 | China | Tinea capitis | Ping Zhan | T. mentagrophytes IX |
| B | XM41 | China | Tinea faciei | Ping Zhan | T. mentagrophytes IX |
| B | XM45 | China | Tinea faciei | Ping Zhan | T. mentagrophytes IX |
| B | XM46 | China | Tinea faciei | Ping Zhan | T. mentagrophytes IX |
| B | XM49 | China | Tinea faciei | Ping Zhan | T. mentagrophytes IX |
| B | XM51 | China | Tinea capitis | Ping Zhan | T. mentagrophytes IX |
| B | XM52 | China | Tinea faciei | Ping Zhan | T. mentagrophytes IX |
| B | XM55 | China | Tinea cruris | Ping Zhan | T. mentagrophytes IX |
| B | 214691/17 | Germany | Tinea corporis | Pietro Nenoff | T. mentagrophytes IX |
| C | 211497/17 | West India | Tinea corporis | Pietro Nenoff | T. mentagrophytes VIII |
| C | 211501/17 | West India | Tinea corporis | Pietro Nenoff | T. mentagrophytes VIII |
| C | 211509/17 | West India | Tinea | Pietro Nenoff | T. mentagrophytes VIII |
| C | 216500/17 | Nord India | Tinea | Pietro Nenoff | T. mentagrophytes VIII |
| C | 216520/17 | Nord India | Tinea | Pietro Nenoff | T. mentagrophytes VIII |
| C | 200074/18 | South India | Tinea | Pietro Nenoff | T. mentagrophytes VIII |
| C | 200095/18 | South India | Tinea | Pietro Nenoff | T. mentagrophytes VIII |
| C | 200100/18 | West India | Tinea corporis | Pietro Nenoff | T. mentagrophytes VIII |
| C | 200101/18 | West India | Tinea corporis | Pietro Nenoff | T. mentagrophytes VIII |
| C | 1663 | West India | unknown | Pietro Nenoff | T. mentagrophytes VIII |
| C | 1681 | West India | Tinea corporis | Pietro Nenoff | T. mentagrophytes VIII |
| C | 1728 | East India | Tinea | Pietro Nenoff | T. mentagrophytes VIII |
| C | 1733 | East India | Tinea | Pietro Nenoff | T. mentagrophytes VIII |
| C | i2 | India | Tinea cruris | Rameshwari Thakur | T. mentagrophytes VIII |
| C | i3 | India | Tinea cruris | Rameshwari Thakur | T. mentagrophytes VIII |
| C | i5 | India | Tinea manuum | Rameshwari Thakur | T. mentagrophytes VIII |
| C | i7 | India | Tinea cruris | Rameshwari Thakur | T. mentagrophytes VIII |
| C | i8 | India | Tinea cruris | Rameshwari Thakur | T. mentagrophytes VIII |
| C | i10 | India | Tinea cruris | Rameshwari Thakur | T. mentagrophytes VIII |
| C | i11 | India | Tinea pedis | Rameshwari Thakur | T. mentagrophytes VIII |
| C | i12 | India | Tinea cruris | Rameshwari Thakur | T. mentagrophytes VIII |
| C | i14 | India | Tinea cruris | Rameshwari Thakur | T. mentagrophytes VIII |
| C | i15 | India | Tinea cruris | Rameshwari Thakur | T. mentagrophytes VIII |
| C | i16 | India | Tinea faciei | Rameshwari Thakur | T. mentagrophytes VIII |
| C | i19 | India | Tinea cruris | Rameshwari Thakur | T. mentagrophytes VIII |
| C | i20 | India | Tinea cruris | Rameshwari Thakur | T. mentagrophytes VIII |
| C | i21 | India | Tinea cruris | Rameshwari Thakur | T. mentagrophytes VIII |
| C | i23 | India | Tinea cruris | Rameshwari Thakur | T. mentagrophytes VIII |
| C | i24 | India | Tinea cruris | Rameshwari Thakur | T. mentagrophytes VIII |
| C | i25 | India | Tinea cruris | Rameshwari Thakur | T. mentagrophytes VIII |
| C | i26 | India | Tinea cruris | Rameshwari Thakur | T. mentagrophytes VIII |
| C | i27 | India | Tinea cruris | Rameshwari Thakur | T. mentagrophytes VIII |
| C | i29 | India | Tinea cruris | Rameshwari Thakur | T. mentagrophytes VIII |
| C | i30 | India | Tinea cruris | Rameshwari Thakur | T. mentagrophytes VIII |
| C | i35 | India | Tinea cruris | Rameshwari Thakur | T. mentagrophytes VIII |
| C | i36 | India | Tinea cruris | Rameshwari Thakur | T. mentagrophytes VIII |
| C | i38 | India | Tinea cruris | Rameshwari Thakur | T. mentagrophytes VIII |
| C | i39 | India | Tinea cruris | Rameshwari Thakur | T. mentagrophytes VIII |
| C | i40 | India | Tinea cruris | Rameshwari Thakur | T. mentagrophytes VIII |
| C | i41 | India | Tinea cruris | Rameshwari Thakur | T. mentagrophytes VIII |
| C | i42 | India | Tinea cruris | Rameshwari Thakur | T. mentagrophytes VIII |
| C | i43 | India | Tinea cruris | Rameshwari Thakur | T. mentagrophytes VIII |
| C | i45 | India | Tinea cruris | Rameshwari Thakur | T. mentagrophytes VIII |
| C | i47 | India | Tinea cruris | Rameshwari Thakur | T. mentagrophytes VIII |
| C | i48 | India | Tinea cruris | Rameshwari Thakur | T. mentagrophytes VIII |
| C | i49 | India | Tinea faciei | Rameshwari Thakur | T. mentagrophytes VIII |
| C | CBS 146623 | Japan | Tine corporis | Rui Kano | T. mentagrophytes VIII |
| C | CBS 146624 | Japan | Tinea corporis | Rui Kano | T. mentagrophytes VIII |
