# Supplementary material for: Taxonomy of the Trichophyton mentagrophytes/T. interdigitale Species Complex Harboring the Highly Virulent, Multiresistant Genotype T. indotineae
Source: Mycopathologia. 2021 Apr 13;186(3):315–26. doi: 10.1007/s11046-021-00544-2 (PMC8249266; doi:10.1007/s11046-021-00544-2)
Supplement: Supplementary file 4 — Supplementary file4 (DOCX 19 kb) [file 11046_2021_544_MOESM4_ESM.docx]

**Table S-2.** Primers used in this study.

| Primer name | Primer sequence (5'–3') | References |
| --- | --- | --- |
| ITS 1 | TCCGTAGGTGAACCTGCGG | [22] |
| ITS 4 | TCCTCCGCTTATTGATATGC | [22] |
| EF1α-F | CACATTAACTTGGTCGTTATCG | [39] |
| EF1α-R | CATCCTTGGAGATACCAGC | [39] |
| HMG-F | AGGGAAACTTCAATTCCATCA | [40] |
| HMG-R | GACAGCGTGAACAGAGTCTATC | [40] |
| alpha-box-F | TCTCCTGCTGCCATGGGAGCT | [40] |
| alpha-box-R | CCATTGGATTGATGTGTGCA | [40] |
