# Supplementary material for: Taxonomy of the Trichophyton mentagrophytes/T. interdigitale Species Complex Harboring the Highly Virulent, Multiresistant Genotype T. indotineae
Source: Mycopathologia. 2021 Apr 13;186(3):315–26. doi: 10.1007/s11046-021-00544-2 (PMC8249266; doi:10.1007/s11046-021-00544-2)
Supplement: Supplementary file 5 — Supplementary file5 (DOCX 16 kb) [file 11046_2021_544_MOESM5_ESM.docx]

**Table S-3. The information of animal strains.**

| Number | ITS genotype | Tef1-α | HMG | Mating type |
| --- | --- | --- | --- | --- |
| CBS 124425 | T. mentagrophytes III* | T. mentagrophytes | T. mentagrophytes | + and - |
| CBS 124421 | T. mentagrophytes III* | T. mentagrophytes | T. mentagrophytes | + and - |
| CBS 124420 | T. mentagrophytes III* | T. mentagrophytes | T. mentagrophytes | + and - |
| CBS 124415 | T. mentagrophytes III* | T. mentagrophytes | T. mentagrophytes | + and - |
| CBS 124410 | T. mentagrophytes III* | T. mentagrophytes | T. mentagrophytes | + and - |
| IHEM 22711 | T. mentagrophytes III* | T. mentagrophytes | T. mentagrophytes | + and - |
| IHEM 22709 | T. mentagrophytes III | T. mentagrophytes | T. mentagrophytes | + |
| IHEM 22712 | T. mentagrophytes III | T. mentagrophytes | T. mentagrophytes | + and - |
| IHEM 22720 | T. mentagrophytes III | T. mentagrophytes | T. mentagrophytes | + |
| IHEM 22727 | T. mentagrophytes III | T. mentagrophytes | T. mentagrophytes | + |
| IHEM 22739 | T. mentagrophytes IV | T. mentagrophytes | T. mentagrophytes | + and - |
| IHEM 22740 | T. mentagrophytes IV | T. mentagrophytes | T. mentagrophytes | + |
| IHEM 10162 | T. mentagrophytes IV | T. mentagrophytes | T. mentagrophytes | + and - |
| V296-57 | T. mentagrophytes IV | T. mentagrophytes | T. mentagrophytes | + and - |
| V296-56 | T. interdigitale | T. interdigitale | T. mentagrophytes | + and - |
| V296-58 | T. interdigitale | T. interdigitale | T. mentagrophytes | + and - |
| IHEM 22714 | T. interdigitale | T. interdigitale | T. interdigitale | + and - |
