# Supplementary material for: Taxonomy of the Trichophyton mentagrophytes/T. interdigitale Species Complex Harboring the Highly Virulent, Multiresistant Genotype T. indotineae
Source: Mycopathologia. 2021 Apr 13;186(3):315–26. doi: 10.1007/s11046-021-00544-2 (PMC8249266; doi:10.1007/s11046-021-00544-2)
Supplement: Supplementary file 6 — Supplementary file6 (DOCX 16 kb) [file 11046_2021_544_MOESM6_ESM.docx]

**Table S-4.** The physiological results.

| Physiological | Results | T. interdigitale | T. mentagrophytes | T. indotineae |
| --- | --- | --- | --- | --- |
| Tween-80 | ‾Χ±σ | 3.25±1.98 | 3.94±1.77 | 1.67±1.2 |
|  | Undetected | 1 | 16 | 0 |
|  | Total | 64 | 69 | 48 |
| Hair perforation | Negative | 1 | 4 | 35 |
|  | Positive | 62 | 49 | 13 |
|  | Undetected | 1 | 16 | 0 |
| Keratin azure | Negative | 3 | 15 | 7 |
|  | Weakly positive | 25 | 33 | 40 |
|  | Positive | 35 | 4 | 1 |
|  | Undected | 1 | 17 | 0 |
| Urease hydrolysis | Negative | 16 | 20 | 35 |
|  | Weakly positive | 11 | 17 | 11 |
|  | Positive | 37 | 24 | 2 |
|  | Undected | 0 | 8 | 0 |
